# Supplementary material for: Phyllanthus emblica: Phytochemistry, Antimicrobial Potential with Antibiotic Enhancement, and Toxicity Insights
Source: Microorganisms. 2025 Mar 6;13(3):611. doi: 10.3390/microorganisms13030611 (PMC11945131; doi:10.3390/microorganisms13030611)
Supplement: Supplementary file 1 [file microorganisms-13-00611-s001.zip › microorganisms-3480523-supplementary.pdf]

Electronic Supplementary Material

Phyllanthus emblica: Phytochemistry, Antimicrobial Potential with Antibiotic Enhancement, and Toxicity Insights

Gagan Tiwana [1], Ian Edwin Cock [2,3] and Matthew James Cheesman [1,*]

[1] School of Pharmacy and Medical Sciences, Gold Coast Campus, Griffith University, Gold Coast 4222, Australia; g.tiwana@griffith.edu.au
[2] School of Environment and Science, Nathan Campus, Griffith University, Brisbane 4111, Australia; i.cock@griffith.edu.au
[3] Centre for Planetary Health and Food Security, Nathan Campus, Griffith University, 170 Kessels Rd., Nathan 4111, Australia
* Correspondence: m.cheesman@griffith.edu.au; Tel.: +61-7-355529230

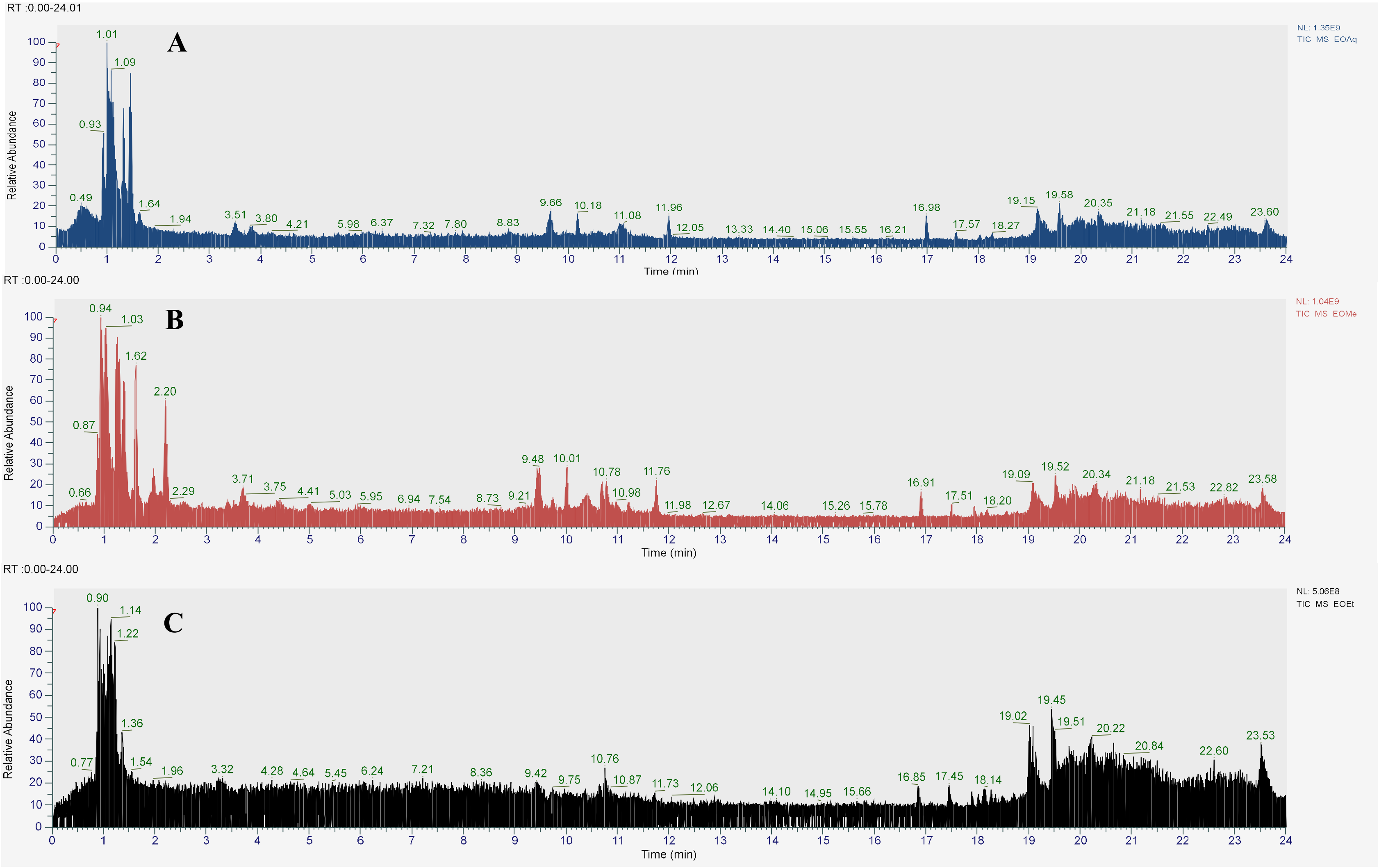

Figure S1. LC-MS total compound chromatograms of (**A**) EO-Aq (*P. emblica* aqueous), (**B**) EO-MeOH (*P. emblica* methanol), and (**C**) EO-EtOAc (*P. emblica* ethyl acetate).

Table S1. LC-MS putative identification and % relative abundance of phytochemicals identified in the aqueous (AQ), methanolic (MeOH), and ethyl acetate (EtOAc) fruit extracts of *P. emblica* using the negative ionisation mode. Compounds less than 0.01% of the total area were considered as trace amounts and denoted as T. Phytochemicals not present in either of the extracts are denoted by (-).

Retention Time (min)	Molecular Weight	Empirical Formula	Putative Compounds	% Relative Abundance		
				Aq	MeOH	EtOAc
1.155	103.0997	$C_5H_{13}NO$	Choline	10.04%	9.28%	-
1.155	174.1115	$C_6H_{14}N_4O_2$	L-Arginine	-	0.11%	-
1.156	182.0789	$C_6H_{14}O_6$	Galactitol	-	0.09%	-
1.159	90.03164	$C_3H_6O_3$	Glyceraldehyde	-	0.46%	2.54%
1.17	342.1159	$C_{12}H_{22}O_{11}$	α, α-Trehalose	-	0.23%	-
1.178	219.1105	$C_9H_{17}NO_5$	Pantothenic acid	-	0.05%	-
1.181	147.053	$C_5H_9NO_4$	L-Glutamic acid	-	0.28%	0.13%
1.187	96.021	$C_5H_4O_2$	2H-Pyran-2-one	-	2.24%	-
1.191	148.037	$C_5H_8O_5$	δ-Ribono-1,4-lactone	-	1.51%	0.68%
1.192	149.0686	$C_5H_{11}NO_4$	4-Amino-4-deoxyarabinose	-	0.01%	-
1.232	141.0424	$C_6H_7NO_3$	2-Aminomuconic acid semialdehyde	-	0.10%	-
1.238	135.0543	$C_5H_5N_5$	Adenine	0.56%	0.27%	-
1.248	142.0264	$C_6H_6O_4$	Sumiki's acid	-	0.30%	-
1.251	160.0369	$C_6H_8O_5$	Cortalcerone	-	1.14%	-
1.253	145.085	$C_5H_{11}N_3O_2$	4-Guanidinobutanoic acid	0.17%	0.17%	-
1.254	86.03666	$C_4H_6O_2$	gamma-Butyrolactone	0.65%	0.85%	-
1.258	174.0526	$C_7H_{10}O_5$	3,4,5-trihydroxycyclohex-1-ene-1-carboxylic acid	0.14%	0.19%	0.27%
1.264	230.0611	$C_{10}H_{14}O_4S$	Thymol sulfate	-	0.02%	-
1.268	161.0685	$C_6H_{11}NO_4$	Aminoadipic acid	-	0.28%	-
1.269	116.0108	$C_4H_4O_4$	Maleic acid	0.75%	0.97%	-
1.292	103.09957	$C_5H_{13}NO$	2-Amino-3-methyl-1-butanol	-	-	0.16%
1.333	92.04719	$C_3H_8O_3$	Glycerol	-	-	0.03%
1.371	194.0789	$C_7H_{14}O_6$	Methyl β-D-glucopyranoside	-	0.09%	-
1.373	356.0377	$C_{14}H_{12}O_{11}$	(+)-Chebulic acid	0.15%	0.95%	2.55%
1.377	158.02104	$C_6H_6O_5$	2-Methylene-4-oxopentanedioic acid	-	-	18.37%
1.406	129.0425	$C_5H_7NO_3$	4-Oxoproline	0.11%	0.09%	0.51%
1.41	100.0523	$C_5H_8O_2$	Tiglic acid	-	0.50%	-
1.433	120.04207	$C_4H_8O_4$	(S)-3,4-Dihydroxybutyric acid	0.17%	-	-
1.454	137.0475	$C_7H_7NO_2$	Trigonelline	2.53%	0.03%	-
1.457	130.02623	$C_5H_6O_4$	Mesaconic acid	0.48%	-	-
1.462	140.0471	$C_7H_8O_3$	Ethyl maltol	-	0.69%	-
1.463	144.04209	$C_6H_8O_4$	5-hydroxy-4-methoxy-5,6-dihydro-2H-pyran-2-one	0.59%	-	-
1.463	128.04715	$C_6H_8O_3$	Osmundalactone	0.10%	-	-
1.464	104.01077	$C_3H_4O_4$	Malonic acid	0.23%	-	-
1.465	160.037	$C_6H_8O_5$	Oxoadipic acid	-	0.22%	0.03%

1.466	129.0424	$C_5 H_7 N O_3$	L-Pyroglutamic acid	-	0.93%	-
1.466	116.04719	$C_5 H_8 O_3$	alpha-Ketoisovaleric acid	0.19%	-	-
1.466	190.09508	$C_7 H_{14} N_2 O_4$	Diaminopimelic acid	0.03%	-	-
1.468	105.04247	$C_3 H_7 N O_3$	L-Serine	0.20%	-	-
1.471	162.01627	$C_5 H_6 O_6$	4-Hydroxy-2-oxoglutaric acid	0.41%	-	-
1.476	115.06315	$C_5 H_9 N O_2$	Proline	1.78%	-	-
1.486	128.0107	$C_5 H_4 O_4$	5-Hydroxy-2-furoic acid	0.17%	0.64%	-
1.491	186.10007	$C_8 H_{14} N_2 O_3$	Alanylproline	0.05%	-	-
1.499	144.05303	$C_5 H_8 N_2 O_3$	N-Nitrosoproline	0.03%	-	-
1.517	125.04753	$C_6 H_7 N O_2$	3-Aminobenzene-1,2-diol	0.14%	-	0.18%
1.534	114.0679	$C_6 H_{10} O_2$	delta-Hexanolactone	-	0.12%	-
1.551	134.02106	$C_4 H_6 O_5$	Malic acid	0.07%	-	-
1.561	167.05769	$C_8 H_9 N O_3$	Pyridoxal	0.01%	-	-
1.587	104.0472	$C_4 H_8 O_3$	2-Hydroxybutyric acid	-	0.17%	-
1.589	206.0424	$C_7 H_{10} O_7$	2-Methylcitric acid	-	0.06%	-
1.593	96.02098	$C_5 H_4 O_2$	2-Furancarboxaldehyde	0.06%	0.07%	0.38%
1.593	90.03157	$C_3 H_6 O_3$	L-(+)-Lactic acid	-	-	0.26%
1.596	98.03668	$C_5 H_6 O_2$	5-Hydroxy-4-pentenoic acid d-lactone	0.14%	0.10%	-
1.597	272.05292	$C_{11} H_{12} O_8$	Fukiic acid	-	-	15.32%
1.619	85.089	$C_5 H_{11} N$	Piperidine	0.06%	0.34%	0.12%
1.62	131.0944	$C_6 H_{13} N O_2$	Isoleucine	0.45%	0.33%	0.19%
1.622	214.1314	$C_{10} H_{18} N_2 O_3$	Valylproline	0.20%	0.22%	-
1.652	204.02678	$C_7 H_8 O_7$	Daucic acid	0.08%	-	-
1.656	130.0265	$C_5 H_6 O_4$	Itaconic acid	-	0.15%	-
1.69	220.0369	$C_{11} H_8 O_5$	Dracunculin	-	0.00%	-
1.752	210.03735	$C_6 H_{10} O_8$	D-Saccharic acid	0.97%	-	8.83%
1.769	192.0269	$C_6 H_8 O_7$	Isocitric acid	15.42%	17.26%	1.61%
1.781	174.0163	$C_6 H_6 O_6$	cis-Aconitic acid	-	0.10%	-
1.803	227.079	$C_{10} H_{13} N O_5$	Pretyrosine	0.33%	0.07%	-
1.809	194.06895	$C_9 H_{10} N_2 O_3$	4-Aminohippuric acid	0.01%	-	-
1.814	192.02685	$C_6 H_8 O_7$	Citric acid	5.62%	-	2.61%
1.933	370.0534	$C_{15} H_{14} O_{11}$	2-O-Caffeoylhydroxycitric acid	-	0.16%	-
1.947	206.02141	$C_{10} H_6 O_5$	Flaviolin	-	-	0.08%
1.968	316.0794	$C_{13} H_{16} O_9$	Ginnalin B	-	0.03%	-
1.993	112.016	$C_5 H_4 O_3$	2-Furoic acid	0.26%	0.06%	-
2.025	87.10478	$C_5 H_{13} N$	Isoamylamine	0.16%	0.17%	-
2.066	134.0215	$C_4 H_6 O_5$	D-(+)-Malic acid	0.38%	2.96%	2.41%
2.067	148.03702	$C_5 H_8 O_5$	Ribonolactone	0.09%	-	-
2.08	266.0427	$C_{12} H_{10} O_7$	2-O-p-Coumaroyltartronic acid	-	0.00%	-
2.084	634.08037	$C_{27} H_{22} O_{18}$	Corilagin	0.30%	-	-

2.088	278.0061	$C_{13} H_{10} O_3 S_2$	Arctic acid C	0.06%	-	-
2.102	326.0637	$C_{14} H_{14} O_9$	Fertaric acid	-	-	0.05%
2.106	165.07881	$C_9 H_{11} N O_2$	L-Phenylalanine	-	-	0.10%
2.118	143.0582	$C_6 H_9 N O_3$	2R-amino-4S-hydroxy-5-hexynoic acid	-	2.34%	-
2.118	115.0632	$C_5 H_9 N O_2$	DL-Allylglycine	-	0.17%	-
2.122	154.02648	$C_7 H_6 O_4$	Gentisic acid	0.02%	-	0.02%
2.132	358.03209	$C_{17} H_{10} O_9$	Distemonanthin	-	-	0.02%
2.155	182.02146	$C_8 H_6 O_5$	2-Hydroxyterephthalic acid	0.03%	-	-
2.165	222.05273	$C_{11} H_{10} O_5$	Isofraxidin	0.01%	-	-
2.18	108.021	$C_6 H_4 O_2$	1,2-Benzoquinone	1.00%	0.51%	-
2.186	178.02655	$C_9 H_6 O_4$	Aesculetin	0.01%	-	0.03%
2.197	143.04048	$C_6 H_9 N O S$	2-Propionyl-2-thiazoline	0.02%	-	-
2.301	173.14141	$C_9 H_{19} N O_2$	4-Piperidinol, 1-hydroxy-2,2,6,6-tetramethyl-	-	-	0.03%
2.303	332.0742	$C_{13} H_{16} O_{10}$	6-Galloylglucose	19.79%	2.80%	5.03%
2.363	139.06323	$C_7 H_9 N O_2$	3,4-Dihydroxybenzylamine	0.01%	-	-
2.375	204.00925	$C_7 H_8 O_5 S$	O-methoxy catechol-O-sulphate	0.01%	-	-
2.415	354.0581	$C_{15} H_{14} O_{10}$	2-O-p-Coumaroylhydroxycitric acid	-	0.37%	-
2.51	381.16362	$C_{15} H_{27} N O_{10}$	(R)-Pantothenic acid 4'-O-β-D-glucoside	0.02%	-	-
2.531	174.0528	$C_7 H_{10} O_5$	Shikimic acid	-	0.00%	-
2.631	240.06348	$C_{11} H_{12} O_6$	Lignicol	0.04%	-	-
2.645	301.18862	$C_{15} H_{27} N O_5$	3-Hydroxy-cis-5-octenoylcarnitine	-	-	0.53%
2.675	496.0849	$C_{21} H_{20} O_{14}$	Hibiscetin 3-glucoside	-	0.01%	-
2.786	346.0902	$C_{14} H_{18} O_{10}$	Methyl 6-O-galloyl-β-D-glucopyranoside	0.01%	0.01%	-
2.884	202.13161	$C_9 H_{18} N_2 O_3$	Alanylisoleucine	0.00%	-	-
2.87	182.0578	$C_9 H_{10} O_4$	Homovanillic acid	-	0.03%	-
2.926	146.0579	$C_6 H_{10} O_4$	2-Methylglutaric acid	-	0.01%	0.06%
3.026	260.1371	$C_{11} H_{20} N_2 O_5$	gamma-Glutamylisoleucine	0.01%	-	-
3.093	102.0316	$C_4 H_6 O_3$	Acetoacetic acid	0.83%	0.13%	0.56%
3.094	155.0218	$C_6 H_5 N O_4$	4-Nitrocatechol	-	0.03%	-
3.096	111.032	$C_5 H_5 N O_2$	Pyrrole-2-carboxylic acid	0.02%	0.02%	-
3.164	228.1473	$C_{11} H_{20} N_2 O_3$	Prolylleucine	0.18%	0.20%	0.28%
3.272	342.09498	$C_{15} H_{18} O_9$	Glucocaffeic acid	-	-	1.02%
3.405	187.0632	$C_{11} H_9 N O_2$	Indoleacrylic acid	0.02%	-	-
3.418	302.0638	$C_{12} H_{14} O_9$	Pyrogallol-2-O-glucuronide	10.94%	4.52%	1.03%
3.48	296.05274	$C_{13} H_{12} O_8$	Caffeoylmalic acid	0.01%	-	-
3.88	185.10482	$C_9 H_{15} N O_3$	Pseudoecgonine	-	-	0.01%
3.914	484.0849	$C_{20} H_{20} O_{14}$	Hamamelitannin	0.35%	0.74%	0.11%
3.919	126.0316	$C_6 H_6 O_3$	Phloroglucinol	1.55%	0.76%	0.79%

4.151	232.00061	$C_{12} H_8 O S_2$	Arctinal	-	-	0.01%
4.236	348.08412	$C_{17} H_{16} O_8$	3,5,7,3',4',5'-Hexahydroxy-6,8-dimethylflavanone	-	-	0.01%
4.558	262.1315	$C_{14} H_{18} N_2 O_3$	5-(tert-butyl)-2-methyl-N-(5-methyl-3-isoxazolyl)-3-furamide	-	0.08%	-
4.584	261.9998	$C_5 H_{12} O_8 P_2$	(2E)-4-Hydroxy-3-methylbut-2-en-1-yl trihydrogen diphosphate	-	0.02%	-
4.585	312.0479	$C_{13} H_{12} O_9$	Caftaric acid	-	1.69%	-
4.638	390.1528	$C_{17} H_{26} O_{10}$	Todatriol glucoside	0.00%	-	-
4.679	484.0857	$C_{20} H_{20} O_{14}$	1,6-Bis-O-(3,4,5-trihydroxybenzoyl) hexopyranose	1.59%	7.35%	3.59%
4.92	328.11577	$C_{15} H_{20} O_8$	Ethylvanillin glucoside	0.00%	-	-
5.112	176.0684	$C_7 H_{12} O_5$	2-Isopropylmalic acid	0.01%	0.01%	-
5.438	484.1219	$C_{21} H_{24} O_{13}$	Diospyrin	0.04%	0.05%	-
6.312	264.027	$C_{13} H_{12} O_2 S_2$	Arctinol	0.00%	0.01%	-
6.739	230.1051	$C_{13} H_{14} N_2 O_2$	(1xi,3xi)-1,2,3,4-Tetrahydro-1-methyl-beta-carboline-3-carboxylic acid	0.02%	0.02%	-
8.389	384.0693	$C_{16} H_{16} O_{11}$	2-O-Feruloylhydroxycitric acid	-	0.06%	-
8.807	450.1162	$C_{21} H_{22} O_{11}$	Hovetrichoside C	-	0.01%	-
8.821	634.0809	$C_{27} H_{22} O_{18}$	Sanguiin H4	-	0.03%	0.77%
9.575	478.0749	$C_{21} H_{18} O_{13}$	6-Hydroxyluteolin 6-glucuronide	-	0.02%	0.01%
9.658	372.10579	$C_{16} H_{20} O_{10}$	Veranisatin C	0.00%	-	-
9.699	126.0317	$C_6 H_6 O_3$	Pyrogallol	-	9.18%	4.74%
9.737	116.0108	$C_4 H_4 O_4$	Fumaric acid	0.07%	0.09%	0.10%
9.848	271.17793	$C_{14} H_{25} N O_4$	4-Oxo-4-[(3-oxo-2-decanyl) amino] butanoic acid	-	-	0.24%
9.898	498.101	$C_{21} H_{22} O_{14}$	Methyl 4,6-di-O-galloyl-β-D-glucopyranoside	-	0.01%	-
10.232	226.1203	$C_{12} H_{18} O_4$	Allixin	0.01%	0.02%	-
10.234	388.1728	$C_{18} H_{28} O_9$	β-D-Glucopyranosyl-11-hydroxyjasmonic acid	-	0.02%	-
10.264	314.0635	$C_{13} H_{14} O_9$	β-D-Glucopyranuronic acid	0.99%	2.18%	-
10.287	299.20928	$C_{16} H_{29} N O_4$	(2Z)-non-2-enoylcarnitine	-	-	0.18%
10.44	636.0962	$C_{27} H_{24} O_{18}$	1,2,6-Trigalloyl-β-D-glucopyranose	-	1.30%	-
10.455	292.02182	$C_{13} H_8 O_8$	Brevifolincarboxylic acid	-	-	0.12%
10.712	480.0902	$C_{21} H_{20} O_{13}$	Telephioidin	-	0.02%	-
10.753	610.1531	$C_{27} H_{30} O_{16}$	Aureusidin 4,6-diglucoside	-	0.01%	-
10.802	368.1103	$C_{17} H_{20} O_9$	3-Feruloylquinic acid	-	0.03%	-
10.872	434.04837	$C_{19} H_{14} O_{12}$	Ellagic acid arabinoside	-	-	0.05%
10.893	126.10438	$C_8 H_{14} O$	(E)-2-octenal	-	-	0.02%
10.925	176.0319	$C_6 H_8 O_6$	Ascorbic acid	-	0.11%	-
10.932	636.09613	$C_{27} H_{24} O_{18}$	1,3,4-Trigalloyl-β-D-glucopyranose	0.02%	-	0.16%
10.932	327.21541	$C_{16} H_{29} N_3 O_4$	Diprotin B	0.02%	-	-
11.058	372.10542	$C_{16} H_{20} O_{10}$	Dihydroferulic acid 4-O-glucuronide	0.05%	-	-
11.131	594.1578	$C_{27} H_{30} O_{15}$	Palasitrin	-	0.02%	-

11.138	326.10017	$C_{15}H_{18}O_8$	Melilotoside	0.04%	-	-
11.151	265.1311	$C_{14}H_{19}NO_4$	N-(2,6-Dimethylphenyl)-N-(methoxyacetyl) alanine	-	0.04%	-
11.19	494.0694	$C_{21}H_{18}O_{14}$	8-Hydroxytricetin 7-glucuronide	-	0.03%	-
11.195	170.0214	$C_7H_6O_5$	Gallic acid	1.19%	0.38%	1.42%
11.221	442.09013	$C_{22}H_{18}O_{10}$	Robinetinidol 3-O-gallate	-	-	0.01%
11.357	368.11067	$C_{17}H_{20}O_9$	3-O-Caffeoyl-1-O-methylquinic acid	0.03%	-	-
11.372	602.1273	$C_{28}H_{26}O_{15}$	(2S) -5,7,3',4'-Tetrahydroxyflavanone 7- (6-galloylglucoside)	-	0.02%	-
11.422	464.0953	$C_{21}H_{20}O_{12}$	Myricitrin	0.14%	0.21%	-
11.435	284.12547	$C_{14}H_{20}O_6$	(2R,3S,4S,5R,6R)-2-(hydroxymethyl)-6-(2-phenylethoxy) oxane-3,4,5-triol	0.07%	-	-
11.602	216.0996	$C_{10}H_{16}O_5$	(4S,5S,8S,10R)-4,5,8-trihydroxy-10-methyl-3,4,5,8,9,10-hexahydro-2H-oxecin-2-one	0.08%	0.08%	0.50%
11.608	180.09368	$C_{14}H_{12}$	Stilbene	-	-	1.04%
11.616	172.0735	$C_8H_{12}O_4$	(-)-Corey lactone	0.03%	0.06%	-
11.628	128.0837	$C_7H_{12}O_2$	1,3-Diacetylpropane	-	0.03%	0.03%
11.641	448.0641	$C_{20}H_{16}O_{12}$	Ellagic acid 2-rhamnoside	0.01%	0.01%	0.06%
11.663	478.11122	$C_{22}H_{22}O_{12}$	6-Methoxyluteolin 7-glucoside	0.01%	-	-
11.687	230.1154	$C_{11}H_{18}O_5$	2-(6-Hydroxyhexyl)-3-methylenesuccinic acid	0.04%	0.08%	-
11.688	186.1255	$C_{10}H_{18}O_3$	3-Oxodecanoic acid	0.04%	0.05%	0.01%
11.729	522.21033	$C_{26}H_{34}O_{11}$	Isolariciresinol 9-O-β-D-glucoside	0.00%	-	-
11.731	600.1113	$C_{28}H_{24}O_{15}$	Isoorientin 2''-O-gallate	0.01%	0.01%	-
11.746	162.06799	$C_{10}H_{10}O_2$	4,5-Dihydro-1-benzoxepin-3(2H)-one	-	-	0.01%
11.753	190.06289	$C_{11}H_{10}O_3$	7-Methoxy-6-methyl-2H-1-benzopyran-2-one	-	-	0.02%
11.924	245.1624	$C_{12}H_{23}NO_4$	2-Methylbutyroylcarnitine	-	-	0.01%
11.916	448.1005	$C_{21}H_{20}O_{11}$	Trifolin	0.59%	0.55%	0.31%
11.917	286.0476	$C_{15}H_{10}O_6$	Kaempferol	-	0.46%	0.14%
11.963	184.037	$C_8H_8O_5$	Methyl gallate	0.24%	0.90%	-
11.965	170.0214	$C_7H_6O_5$	2,4,6-Trihydroxybenzoic acid	0.24%	1.69%	1.27%
11.978	310.1049	$C_{15}H_{18}O_7$	(E)-1-O-Cinnamoyl-β-D-glucose	-	3.86%	2.01%
11.979	162.0526	$C_6H_{10}O_5$	2-Hydroxyadipic acid	0.14%	0.15%	0.03%
11.99	302.0062	$C_{14}H_6O_8$	Ellagic acid	12.37%	7.84%	9.17%
11.991	302.0423	$C_{15}H_{10}O_7$	Quercetin	0.18%	0.27%	-
12.033	594.101	$C_{29}H_{22}O_{14}$	Epicatechin 3,5-di-O-gallate	-	0.00%	-
12.063	216.15115	$C_{15}H_{20}O$	2-Hexyl-3-phenyl-2-propenal	-	-	0.04%
12.179	434.1213	$C_{21}H_{22}O_{10}$	Hemiphloin	-	0.04%	-
12.179	272.0683	$C_{15}H_{12}O_5$	Naringeninchalcone	0.04%	0.06%	-
12.186	326.15141	$C_{20}H_{22}O_4$	2',4',6'-Trihydroxy-3'-prenyldihydrochalcone	0.01%	-	-
12.249	568.1434	$C_{25}H_{28}O_{15}$	5,7,3',5'-Tetrahydroxy-3,6,8,4'-tetramethoxyflavone 3'-glucoside	-	0.00%	-

12.273	302.0794	$C_{16}H_{14}O_6$	2,6,3'-Trihydroxy-4'-methoxy-2-benzylcoumaranone	-	0.00%	-
12.286	504.1842	$C_{22}H_{32}O_{13}$	(S)-Multifidol 2-[apiosyl-(1->6)-glucoside]	0.02%	0.03%	-
12.292	492.0908	$C_{22}H_{20}O_{13}$	6-Methoxyluteolin 7-glucuronide	0.01%	0.03%	1.01%
12.307	286.04732	$C_{15}H_{10}O_6$	Fisetin	0.31%	-	-
12.324	148.0524	$C_9H_8O_2$	Cinnamic acid	1.01%	1.26%	1.45%
12.385	90.03159	$C_3H_6O_3$	L-Lactic acid	0.01%	-	-
12.385	144.04204	$C_6H_8O_4$	3-Hexenedioic acid	0.02%	-	-
12.397	554.2368	$C_{27}H_{38}O_{12}$	Ssioriside	-	0.00%	-
12.437	349.19973	$C_{18}H_{27}N_3O_4$	Coutaric acid	0.02%	-	-
12.464	474.07948	$C_{22}H_{18}O_{12}$	Chicoric acid	0.01%	-	-
12.48	292.09429	$C_{15}H_{16}O_6$	(S)-Angelicain	0.01%	-	-
12.545	432.1059	$C_{21}H_{20}O_{10}$	Afzelin	-	0.03%	-
12.567	434.12115	$C_{21}H_{22}O_{10}$	4H-1-Benzopyran-4-one, 6-β-D-glucopyranosyl-2,3-dihydro-5,7-dihydroxy-2-(4-hydroxyphenyl)-, (S)-	0.03%	-	-
12.674	550.1689	$C_{26}H_{30}O_{13}$	Licuroside	-	0.01%	0.01%
12.739	140.1201	$C_9H_{16}O$	(3Z,6Z)-3,6-Nonadien-1-ol	-	0.00%	-
12.74	228.0997	$C_{11}H_{16}O_5$	Propyl propane thiosulfonate	0.01%	0.02%	-
12.757	150.06792	$C_9H_{10}O_2$	Hydrocinnamic acid	-	-	0.04%
12.769	288.06334	$C_{15}H_{12}O_6$	2,6,3',4'-Tetrahydroxy-2-benzylcoumaranone	0.02%	-	-
12.919	185.14141	$C_{10}H_{19}NO_2$	1-Methylpiperidin-4-yl butanoate	-	-	0.09%
12.937	304.0581	$C_{15}H_{12}O_7$	Nigrescin	0.02%	0.02%	-
12.94	342.1465	$C_{20}H_{22}O_5$	Brosimacutin C	-	0.01%	-
12.983	572.1901	$C_{29}H_{32}O_{12}$	Amorphigenin O-glucoside	0.02%	0.02%	-
12.996	262.01149	$C_{13}H_{10}O_2S_2$	Arctinone A	0.01%	-	-
13.009	262.0477	$C_{13}H_{10}O_6$	Maclurin	-	0.00%	-
13.026	444.1054	$C_{22}H_{20}O_{10}$	3'-O-Methylderhamnosylmaysin	0.08%	0.06%	0.01%
13.038	357.25139	$C_{19}H_{35}NO_5$	(9Z)-3-Hydroxydodecenoylcarnitine	-	-	0.02%
13.043	524.15321	$C_{24}H_{28}O_{13}$	Barbatoflavan	-	-	1.01%
13.046	538.16926	$C_{25}H_{30}O_{13}$	Lippioside I	0.00%	-	-
13.058	218.05794	$C_{12}H_{10}O_4$	Liqcoumarin	0.00%	-	-
13.109	372.14204	$C_{17}H_{24}O_9$	Citrusin E	0.02%	-	-
13.125	186.0891	$C_9H_{14}O_4$	5-(2-Methylpropyl) tetrahydro-2-oxo-3-furancarboxylic acid	-	0.00%	-
13.159	506.1062	$C_{23}H_{22}O_{13}$	Quercetin 3- (6''-ethylglucuronide)	-	0.04%	-
13.188	504.34447	$C_{30}H_{48}O_6$	Protobassic acid	-	-	0.02%
13.188	486.334	$C_{30}H_{46}O_5$	Bassic acid	-	-	0.01%
13.369	462.1164	$C_{22}H_{22}O_{11}$	6-O-[(2E)-3-Phenyl-2-propenoyl]-1-O-(3,4,5-trihydroxybenzoyl)-β-D-glucopyranose	0.32%	0.46%	0.11%
13.43	302.0425	$C_{15}H_{10}O_7$	Bracteatin	-	0.03%	0.05%

13.467	614.1275	$C_{29}H_{26}O_{15}$	6-Cinnamoyl-1,2-digalloylglucose	-	0.09%	-
13.49	312.0999	$C_{18}H_{16}O_5$	3,4-Methylenedioxy-2',4'-dimethoxychalcone	-	0.00%	-
13.561	400.1155	$C_{21}H_{20}O_8$	Torosaflavone A	0.02%	0.03%	-
13.597	308.0793	$C_{17}H_{12}N_2O_4$	Flazin	-	0.02%	-
13.647	462.1162	$C_{22}H_{22}O_{11}$	Leptosin	0.00%	0.03%	0.01%
13.665	596.153	$C_{30}H_{28}O_{13}$	(2S) -5,7,3',4'-Tetrahydroxyflavanone 7- (6-p-coumaroylglucoside)	-	0.03%	-
13.674	370.1052	$C_{20}H_{18}O_7$	2'-Hydroxy-5',6'-dimethoxy-3,4-methylenedioxyfurano [2'',3'':4',3'] dihydrochalcone	-	0.01%	-
13.714	227.0944	$C_{14}H_{13}NO_2$	Koenoline	0.00%	0.00%	-
13.722	274.08384	$C_{15}H_{14}O_5$	Phloretin	0.02%	-	-
13.784	158.05777	$C_7H_{10}O_4$	Succinylacetone	-	-	0.02%
13.874	614.1273	$C_{29}H_{26}O_{15}$	2-Cinnamoyl-1,6-digalloyl-β-D-glucopyranose	-	0.01%	-
13.92	260.15199	$C_{15}H_{20}N_2O_2$	Baptifoline	0.00%	-	-
14.06	506.1061	$C_{23}H_{22}O_{13}$	6-Methoxyluteolin 7-glucuronide methyl ester	-	0.01%	-
14.054	142.06281	$C_7H_{10}O_3$	5-Ethyl-3-hydroxy-4-methyl-2(5H)-furanone	0.01%	-	-
14.278	148.0523	$C_9H_8O_2$	p-Coumaraldehyde	0.18%	0.26%	-
14.34	376.1882	$C_{22}H_{24}N_4O_2$	N-{(3S,5S)-1-Methyl-5-[3-(4-methylphenyl)-1,2,4-oxadiazol-5-yl]-3-pyrrolidinyl}-2-phenylacetamide	0.06%	0.07%	0.11%
14.423	210.0925	$C_8H_{18}O_4S$	2-Ethyl-1-hexanol sulfate	0.02%	0.03%	0.05%
14.472	290.0788	$C_{15}H_{14}O_6$	Marshrin	-	0.04%	-
14.492	390.1677	$C_{21}H_{26}O_7$	(2S,4S,6S)-2-[2-(4-Hydroxy-3-meyhoxyphenyl) ethyl]tetrahydro-6-(4,5-dihydroxy-3-methoxyphenyl)-2H-pyran-4-ol	-	0.01%	-
14.671	200.1412	$C_{11}H_{20}O_3$	(S)-9-Hydroxy-10-undecenoic acid	0.00%	0.00%	0.00%
14.812	286.0477	$C_{15}H_{10}O_6$	Maritimetin	-	0.08%	-
14.829	580.1582	$C_{30}H_{28}O_{12}$	Naringenin 7- (2-p-Coumaroylglucoside)	0.08%	-	-
14.958	272.06837	$C_{15}H_{12}O_5$	Butein	0.03%	-	-
15.062	580.15808	$C_{30}H_{28}O_{12}$	4,2',3',4'-Tetrahydroxychalcone 4'-O-(2''-O-p-coumaroyl) glucoside	-	-	0.04%
15.295	696.40846	$C_{37}H_{60}O_{12}$	Momordicoside E	-	-	0.02%
16.15	316.13099	$C_{18}H_{20}O_5$	4,2',6'-Trihydroxy-4'-methoxy-3',5'-dimethyldihydrochalcone	-	-	1.01%
16.178	520.122	$C_{24}H_{24}O_{13}$	5,2'-Dihydroxy-7,8,6'-trimethoxyflavone 2'-glucuronide	-	0.01%	-
16.602	301.22498	$C_{16}H_{31}NO_4$	2,6 Dimethylheptanoyl carnitine	-	-	0.00%
17.04	268.19374	$C_{18}H_{24}N_2$	N-(1,3-Dimethylbutyl)-N'-phenyl-p-phenylenediamine	-	-	0.41%

17.041	184.09993	$C_{12}H_{12}N_2$	Harmalan	-	-	0.02%
17.047	252.2085	$C_{16}H_{28}O_2$	7Z,10Z-Hexadecadienoic acid	-	0.01%	-
17.048	270.219	$C_{16}H_{30}O_3$	3-Oxohexadecanoic acid	0.00%	0.01%	-
17.051	288.2301	$C_{16}H_{32}O_4$	(S)-10,16-Dihydroxyhexadecanoic acid	0.02%	0.03%	0.26%
17.177	470.3394	$C_{30}H_{46}O_4$	Glycyrrhetinic acid	-	0.02%	-
17.419	161.0476	$C_9H_7NO_2$	2-Indolecarboxylic acid	-	0.00%	-
17.427	170.1305	$C_{10}H_{18}O_2$	trans-Dec-2-enoic acid	0.04%	0.05%	0.13%
17.428	214.1204	$C_{11}H_{18}O_4$	5-Hexyltetrahydro-2-oxo-3-furancarboxylic acid	-	0.05%	-
17.449	822.4026	$C_{42}H_{62}O_{16}$	Glycyrrhizin	0.00%	-	-
17.512	286.12056	$C_{17}H_{18}O_4$	4-Hydroxy-2',4'-dimethoxydihydrochalcone	0.00%	-	-
17.546	156.11483	$C_9H_{16}O_2$	4-Hydroxynonenal	-	-	0.01%
17.682	328.2249	$C_{18}H_{32}O_5$	Corchorifatty acid F	0.01%	-	-
17.76	210.03747	$C_6H_{10}O_8$	Galactaric acid	0.01%	-	-
17.761	148.03709	$C_5H_8O_5$	Citramalic acid	0.00%	-	-
17.86	254.15163	$C_{14}H_{22}O_4$	(2R,5R,6R)-3-[(1E,3E)-hepta-1,3-dien-1-yl]-5,6-dihydroxy-2-(hydroxymethyl) cyclohexan-1-one	-	-	0.30%
17.969	256.07342	$C_{15}H_{12}O_4$	Isoliquiritigenin	-	-	0.01%
18.036	314.11518	$C_{18}H_{18}O_5$	4'-Hydroxyenterolactone	-	-	0.06%
18.608	242.09407	$C_{15}H_{14}O_3$	2',4'-Dihydroxydihydrochalcone	-	-	0.03%
18.634	194.09403	$C_{11}H_{14}O_3$	(R)-3-Hydroxy-5-phenylpentanoic acid	-	-	0.06%
18.635	162.06789	$C_{10}H_{10}O_2$	4-Methoxycinnamaldehyde	-	-	0.12%
18.835	388.20942	$C_{19}H_{32}O_8$	5a,6a-Epoxy-7E-megastigmene-3a,9e-diol 3-glucoside	-	-	0.08%
18.835	268.13078	$C_{14}H_{20}O_5$	Kamahine C	0.01%	-	-
19.009	284.16197	$C_{15}H_{24}O_5$	Alpha-dihydroartemisinin	-	-	0.09%
19.365	240.1146	$C_{16}H_{16}O_2$	1-Acetoxy-4,6-tetradecadiene-8,10,12-triyne	-	0.11%	-
19.366	212.1197	$C_{15}H_{16}O$	11,12-Dihydrolactaroviolin	-	0.03%	-
19.444	184.12494	$C_{14}H_{16}$	1,4-Dimethyl-7-ethylazulene	-	-	0.05%
19.444	240.1147	$C_{16}H_{16}O_2$	3,3',5,5'-Tetramethyldiphenoquinone	-	-	0.94%
19.445	156.09369	$C_{12}H_{12}$	2,6-Dimethyl-naphtalene	-	-	0.04%
19.447	226.099	$C_{15}H_{14}O_2$	7-Hydroxyflavan	-	0.03%	0.02%
19.518	170.10939	$C_{13}H_{14}$	1,6,7-Trimethylnaphthalene	-	-	0.04%
19.52	198.10417	$C_{14}H_{14}O$	Dibenzyl ether	-	-	0.10%
22.747	622.44404	$C_{36}H_{62}O_8$	(20R)-Ginsenoside Rh2	-	-	0.15%
**Grand Total**				100.0%	100.0%	100.0%
